# Remote Monitoring and Holistic Care of Home-Isolated COVID-19 Positive Healthcare Workers Through Digital Technology During the Omicron (B1.1.529) Wave: A Prospective Cohort Study From India

**DOI:** 10.3389/fpubh.2022.936000

**Published:** 2022-07-13

**Authors:** Siddharth Jain, Amit Agarwal, Anupriya Bhardwaj, PVM Lakshmi, Manvi Singh, Anil Chauhan, Meenu Singh

**Affiliations:** ^1^Department of Telemedicine, Postgraduate Institute of Medical Education and Research, Chandigarh, India; ^2^Department of Community Medicine, Postgraduate Institute of Medical Education and Research, Chandigarh, India; ^3^Department of Pediatrics, Postgraduate Institute of Medical Education and Research, Chandigarh, India

**Keywords:** COVID-19, healthcare worker (HCW), digital technology, remote monitoring, cohort

## Abstract

**Background:**

Remote monitoring through digital technology offers a promising solution for the diverse medical, psychological and social issues that plague patients with COVID-19 under home-isolation, but remain neglected due to a lack of streamlined medical services for these patients.

**Methods:**

This prospective cohort study determined the feasibility of remote telemonitoring of healthcare workers with mild COVID-19 under home isolation during the Omicron (B1.1.529) wave and characterized their clinico-demographic profile. A holistic monitoring model comprising of mandatory phone calls at the beginning and end of isolation, assisted by home oximetry, predesigned google forms, and opt-in software-based (eSanjeevani OPD) teleconsultation was employed. Factors associated with development of symptomatic disease were also determined.

**Results:**

Out of 100 COVID-19 positive healthcare workers under home-isolation, data for 94 participants was available [median age 27(20–52) years, 56(60%) females]. 93(99%) patients were previously vaccinated for COVID-19 (median time from last dose = 248 days); 34(36%) had a past history of COVID-19. Fever (67%), myalgia (69%), sore throat/dry cough (70%), and running nose (45%) were the most common symptoms. No patient progressed to moderate-severe disease or required care escalation during the remote monitoring period. Most participants reported several additional psychosocial concerns which were adequately addressed. Symptomatic patients had higher BMI (24.1 vs. 21.8kg/m^2^, *p* = 0.01) compared to asymptomatic patients. Age, past infection with COVID-19, and time since last vaccine dose were not different between symptomatic and asymptomatic patients.

**Conclusion:**

COVID-19 patients under home isolation have multi-faceted medical and psychosocial issues which can be holistically managed remotely through digital technology.

## Introduction

With repeated waves due to newly emerging variants of the novel Severe Acute Respiratory Syndrome-Corona Virus-2 (SARS-CoV-2), the scourge of coronavirus disease 2019 (COVID-19) continues unabated. Two years after being declared a pandemic by the World Health Organization, about 405 million confirmed COVID-19 cases, including ~6 million deaths have been reported as of 11 February 2022 (1). The vast majority (80-90%) of affected patients have asymptomatic or mild disease and can safely be managed under home-isolation without the need for hospital admission. However, despite a “clinically mild” disease, the physical symptoms may sometimes be very disabling and often remain inadequately addressed due to a lack of streamlined medical services for these patients living under a strict home-isolation. A few of these patients have the potential to progress to moderate-or-severe disease, and this transition needs to be picked up early in order to improve patient outcomes. Additionally, the disease or diagnosis itself is associated with a significant stress, phobia, depression and anxiety which continue to persist as neglected issues (2, 3). Concerns about fitness to re-join work or duty, ending of isolation, requirement (if any) of repeat testing prior to it, effect of the infection on vaccine schedules, and management of household contacts can be a cause of significant botheration to these patients. Thus, dedicated mechanisms need to be put in place for a holistic management of these patients' medical, psychological and social issues. Implementation of home monitoring programs or “virtual hospitals”, where patients under home isolation are monitored remotely, offer a promising solution, although the available data are limited and focussed mainly on medical issues and “admission avoidance” (4–12). The mental and psychosocial issues remain largely unaddressed. There is no data on remote monitoring for COVID-19 patients under home-isolation from the Indian subcontinent.

Direct-to-consumer telemedicine also helps to avoid the risk of transmission of COVID-19 to frontline healthcare workers, who are already over-worked and under-staffed due to the infrastructural crises imposed by COVID-19 (7). Healthcare workers affected with COVID-19 face additional challenges when compared to the general population—besides a greater risk of becoming infected, harboring a higher virological burden, and a potentially more serious illness (due to continual exposure), they are prone to have additional work-related and psychosocial stressors (2). Since they form the backbone of healthcare systems which are already working at strained capacities during the COVID-19 pandemic, focussing on their medical and psychosocial needs is of paramount importance.

Finally, although the third wave of the pandemic [epidemiologically linked to the Omicron (B.1.1.529) variant] was deemed to be milder compared to the previous waves (13), the clinico-demographic profile of Indian patients affected during this wave, and the factors associated with development of symptomatic disease remain to be characterized in detail (14).

In an attempt to address these knowledge lacunae, we sought to determine the feasibility of remote telemonitoring and holistic telecare of healthcare workers affected with mild COVID-19 and home-isolated during the third wave of the COVID-19 pandemic, and characterized the clinico-demographic profile of these patients.

## Methods

### Study Design, Setting and Participants

This single-center, prospective cohort study was done at the Postgraduate Institute of Medical Education and Research, a tertiary care center in North India. Consecutive healthcare workers who tested positive for COVID-19 [microbiologically confirmed by SARS-CoV-2 reverse transcriptase polymerase chain reaction (RT-PCR) positivity] between 31 December, 2021 and 28 January, 2022 and were advised home isolation were included after a verbal, informed consent. Those requiring hospital admission for supplemental oxygen or any other reason, those unwilling to give consent to participate in the study, or those whose provided contact details were incorrect, were excluded. A sample size of convenience, with consecutive sampling, was chosen. The study was approved by the Institutional Ethics Committee (INT/IEC/2022/SPL-281) and the principles of the 1964 Helsinki declaration were adhered to.

### Study Procedures

Remote monitoring of COVID-19 positive healthcare workers isolating at home was done under the Department of Telemedicine. Affected healthcare workers were assessed telephonically through fixed-line telephones at baseline (date of positivity) and at day 7 [end of isolation period as per the revised National guidelines (15)] regarding clinical symptoms and oximetry readings. Red flag features (if any) were identified, and concerns related to symptoms, treatment, duration of isolation, repeat testing, testing of household contacts, and re-joining of duty were addressed in consultation with the specialist physician (psychosocial concerns were self-reported by the patient). For all included subjects, baseline demographic data including age, gender, body mass index (BMI), cadre (resident, faculty, nursing staff, student, clerical staff, or research staff), comorbidities, and clinical data relevant to COVID-19 [past COVID-19 infection, number of vaccine doses, type of vaccine received (BBV152/Covaxin or AZD1222/ChAdOx1 nCoV-19/Covishield), and time since the last vaccine dose] were recorded through a google form which was circulated through email or WhatsApp (self-administered), or administered telephonically by the research assistant or telemedicine operator. In addition, telephonic helplines and audio-video teleconsultation services through eSanjeevani OPD (a doctor-to-patient telemedicine system deployed nationally by the Ministry of Health and Family Welfare, Government of India for providing teleconsultations under the Ayushman Bharat Digital Mission) were also put in place for use by the affected patients anytime during their period of isolation. The former were direct access, while the latter required access through one-time password protected sign in on the website or smart phone application. Information about these facilities was notified on the institute website, notice boards and individually to affected patients. Symptomatic treatment was advised to all patients; no antiviral or immunomodulatory therapy was suggested as per the national guidelines, since all patients under home isolation had mild disease (15).

The human resource required for implementation of this model included a team of two medical consultants, a research assistant, a telemedicine operator, and a data entry operator. Phone calls through fixed-line telephones at baseline and day 7 were made by the research assistant and the telemedicine operator, and patients' medical and psychosocial concerns were addressed in consultation with the specialist physician(s). In addition, the medical consultants also went through the completed google forms circulated to the patient for identifying any red flag feature not self-reported by the patient. A psychiatrist was available on-call basis, in case specialist help was required. All staff were already employed and working in the Department of Telemedicine, making the model cost-effective and sustainable. The time taken per consult varied from 5–15 min, depending on the participant's concerns.

### Outcomes

The feasibility of remote monitoring and holistic telecare of home-isolated COVID-19 positive healthcare workers through telemedicine was assessed. In addition, the clinico-demographic profile of the affected healthcare workers was characterized, and the factors associated with the development of symptomatic (vs. asymptomatic) COVID-19 were explored.

### Virological Testing

Nasopharyngeal and throat swabs were collected in viral transport media, transported in cold chain to designated laboratories in the Department of Virology, and used for viral RNA extraction (QiaAmp Viral RNA isolation kit, Hilden, Germany). The extracted RNA was subjected to real time RT-PCR. Variant analysis was not done. Repeat virological testing to document cure, or mark the end of isolation was not done as per the national guidelines (15).

### Statistical Analysis

Data were entered in an excel spreadsheet and analyzed using the Statistical Package for Social Sciences version 26 [SPSS Inc., Chicago, IL]. Normality was tested using the Kolmogorov-Smirnov test. Continuous variables were summarized as mean and standard deviation (normally distributed), or median (range) (non-normal). Categorical variables were assessed as frequencies and proportions. Student's *T*-test or Mann-Whitney U test were used for comparison of means, while proportions were compared using the Chi-square or Fisher's Exact test. All statistical tests were two-sided and a *p*-value cut-off of ≤ 0.05 was used for defining statistical significance.

## Results

Out of a total of 100 healthcare workers who were identified to be COVID positive and telephonically contacted, three did not respond, one did not give consent, and the provided contact number was incorrect for two patients. Thus, the data of total 94 participants were included in the final analysis. Apart from the mandatory telephonic contact at baseline and day 7, 32 additional contacts (30 through telephonic helplines and two *via* eSanjeevani OPD) were initiated by the study patients with the remote monitoring team.

### Demographic Data

The median age of the included participants was 27 (20–52) years. Fifty six (60%) were females. Mean BMI was 23.8 (3.2) kg/m^2^. Twelve (13%) patients reported having one or more comorbidities, most commonly hypertension or lung disease (including bronchial asthma). 53 (56%) of the study participants were medical residents at various stages of their residency, 14 (15%) were nursing staff, 9 (10%) were students, while the remaining were faculty, research, or clerical staff (Table 1).

**Table 1:** Baseline demographic and clinical parameters of the included patients.

**Parameter**	**Value (*n* = 94)**
Age, years	27 (20-52)
Female gender	56 (60%)
Body mass index, kg/m^2^	23.8 (3.2)
**Cadre**	
∙ Faculty	7(7.4%)
∙ Resident	53(56.4%)
∙ Nursing staff	14(14.9%)
∙ Student	9(9.6%)
∙ Clerical staff	5(5.3%)
∙ Research staff	6(6.4%)
**Comorbidities**	
∙ Diabetes	1(1.1%)
∙ Hypertension	4(4.3%)
∙ Kidney disease	1(1.1%)
∙ Lung disease including bronchial asthma	4(4.2%)
∙ Thyroid disease	2(2.1%)
**Vaccine received**	
∙ BBV152/Covaxin	6(6.4%)
∙ AZD1222/ChAdOx1 nCoV-19/Covishield	87(92.6%)
∙ None	1 (1.1%)
**Number of vaccine doses received**	
∙ None	1(1.1%)
∙ One	2(2.1%)
∙ Two	89(94.7%)
∙ Three (including booster)	2(2.1%)
Time since last vaccine dose (days)	248 (9-357)
Past history of COVID-19 infection	
∙ Once	32(34%)
∙ Twice	2(2.1%)

### Clinical Presentation

Eighty one (86%) participants suffered from mild COVID-19, remaining 13 (14%) were asymptomatic. Fever (67%), myalgia or body aches (69%), sore throat or dry cough (70%), and running nose (45%) were the most common symptoms reported. Gastrointestinal symptoms, anosmia, and retro-orbital pain were rare and seen in one patient each. Mean pulse rate and oxygen saturation on pulse oximeter were 89 (16) per minute and 98 (1) %, respectively (Table 2).

**Table 2:** Clinical details of the current COVID-19 infection in the included patients.

**Parameter**	**Value (*n* = 94)**
**Disease severity**
Asymptomatic	13 (14%)
Mild	81 (86%)
Moderate	0
Severe	0
**Clinical presentation**
Fever	63 (67%)
Sore throat or cough	66 (70%)
Running nose	42(45%)
Myalgia or bodyaches	65(69%)
Headache	4(4.3%)
Subjective breathing difficulty	4(4.3%)
Diarrhea	1(1.1%)
Nausea or vomiting	1(1.1%)
Loss of appetite	1(1.1%)
Anosmia	1(1.1%)
Retro-orbital pain	1(1.1%)
**Oximetric readings**
Pulse rate (per minute)	89 (16)
Oxygen saturation on room air (%)	98 (1)

### Need for Care Escalation or Hospitalization

No patient progressed to moderate or severe disease, or required care escalation/hospital admission during the period of telemonitoring during home isolation.

### Psychosocial Concerns

Most participants had additional concerns related to testing and management of family and household contacts including elderly and children, timing of joining back to duty and whether repeat testing was needed prior to it, effect of the infection on vaccine schedules, etc which were mentally bothering them. These were adequately addressed through our integrated remote monitoring model.

### COVID-19 Vaccination and Past Infection With COVID

All patients (except one) were previously vaccinated for COVID-19 using AZD1222 (93%) or BBV152 (7%). Most patients (95%) had completed their two-dose vaccine schedule at the time of being infected with COVID-19, with the median time from the last vaccine dose to acquisition of current COVID-19 being 248 days. Two (2.1%) patients developed COVID-19 even after having received the booster or precautionary third dose; however this was within nine days of immunization with the booster dose. Thirty four (36%) patients had a past history of being infected with COVID-19; two (2%) of these had suffered from COVID-19 twice prior to the current episode (Table 1).

### Factors Associated With Symptomatic COVID-19

Symptomatic patients in the cohort had higher BMI (24.1 vs. 21.8 kg/m^2^, *p* = 0.01) and were more likely to be males (44% vs. 15%, *p* = 0.067) compared to asymptomatic patients (Table 3). There was no difference in age between the two groups. Past infection with COVID-19 and time since last vaccine dose were also not different between symptomatic and asymptomatic patients (Table 3).

**Table 3:** Risk factors for symptomatic COVID-19 in the third wave among healthcare workers.

**Parameter**	**Symptomatic** **(*n* = 81)**	**Asymptomatic** **(*n* = 13)**	***p*** **-value**
Age, years	27 (20-52)	25 (22-52)	0.33
Male gender	36 (44%)	2 (15%)	0.067
Body mass index, kg/m^2^	24.1 (3.2)	21.8 (2.8)	0.01
Presence of comorbidities	12 (15%)	0	0.21
Time since last vaccine dose, days	237 (9-357)	265 (58-321)	0.35
Past COVID-19 infection	32 (40%)	2 (15%)	0.12

## Discussion

This study reports on the success of remote monitoring and holistic care of healthcare workers affected with mild COVID-19 and residing under home isolation through the use of digital technology and characterizes the clinico-demographic profile of these patients infected during the third wave of the pandemic in India caused by the Omicron (B.1.1.529) variant.

The use of virtual care in health management has assumed greater relevance and significance in the current COVID-19 era. The existing healthcare infrastructure and manpower have the potential to be overwhelmed by the huge number of COVID-19 affected patients unless mechanisms for triaging these patients are put in place; since the vast majority of COVID-19 patients have a mild disease, strengthening of virtual care, remote telemonitoring and telecare can help rationalize the scarce healthcare resources. Studies done so far have showed promising results of remote home monitoring for COVID-19 in a pre-hospital as well as post-hospital, step-down setting, with earlier identification of a need for hospitalization or re-admission (in discharged patients), reduction of bed days, and length of hospital stay (4–12). However, most of these studies have included a mix of mild, moderate, or even severe patients in a community setting, and focussed primarily on the medical aspects of a largely “admission avoidance” centric model aimed at reducing the burden on healthcare systems. A significant proportion of suspect (unconfirmed or non-COVID) patients have often been included, compromising the external validity. Our study is different in its focus on providing holistic care for not just medical but also mental and psychosocial needs of patients affected with mild (or even asymptomatic) COVID-19, inclusion of health care workers as the target population rather than the general community as their medical and psychosocial needs may be different, and inclusion of only microbiologically confirmed COVID-19 cases into the study. Care must be taken to avoid extrapolation of data available from different settings since significant ethno-geographic differences in COVID-19-related clinical behavior exist, which could influence the success (or failure) of implementation of remote telemonitoring models, besides the model characteristics themselves. To the best of our knowledge, this is the first study on remote monitoring of mild COVID-19 patients under home isolation from India. Remote monitoring models till date have variably employed smartphone or web applications, in-house softwares, paper-based monitoring, or phone calls, with or without home oximetry, for the purpose of monitoring. Software or application-based approaches are believed to have a wider reach, while phone call-based approaches are considered more inclusive since they are able to overcome barriers associated with technological availability and technological literacy (7). In this study, we used a comprehensive, synergistic model comprising of mandatory phone calls at the beginning and end of isolation, assisted by google forms, home oximetry, and voluntary (opt-in) web or application-based interactions as and when deemed necessary by the patient. This enabled us to overcome limitations of individual approaches and accommodate wider patient preferences. The use of existing resources and staff at the Department of Telemedicine helped to avoid issues pertaining to reallocation of limited resources or staff, cost-effectiveness and sustainability of the model.

Healthcare workers are indispensable to the functioning of healthcare systems. Clear strategies to manage exposed and infected healthcare workers including risk stratification, appropriate clinical monitoring, easy diagnostic access, decision making about quarantine leave and return to work, and home-isolation vs. hospital quarantine are essential to ensure effective staff management and workplace trust (16). Healthcare workers, especially those directly involved in diagnosing or treating suspected or confirmed COVID-19 patients, live in a constant state of psychological stress due to the fear of contracting and transmitting the virus, repeated exposures, and the unpredictable behavior of the disease. This has been shown to result in a higher prevalence of depression, anxiety, sleep disturbances and post-traumatic stress disorders in this population, often necessitating psychosocial intervention (2). Otherwise also, social isolation has been linked to depression even in a non-COVID setting (17). Through remote monitoring, we could manage the home-isolated health care workers both medically and psychologically. None of the study patients progressed clinically to moderate-severe disease, or required care escalation during the period of remote monitoring. Most participants had additional psychosocial concerns which were mentally bothering them; these were adequately addressed through our integrated remote monitoring model. We could not find any study focussing on, or addressing these non-medical aspects of COVID-19.

Our study cohort had milder symptoms compared to studies on remote monitoring from other parts of the world; one of the reasons contributing to this difference, apart from the ethno-geographic differences, was the timing of conduct of this study during the third wave of the pandemic in India, which was epidemiologically linked to Omicron (B.1.1.529), an inherently milder variant of SARS-CoV-2 (14). The clinical presentation was dominated by fever, sore throat, running nose and myalgia. Anosmia, gastrointestinal symptoms, or breathing difficulty were hardly seen, in contrast to the previous waves of the pandemic caused by other SARS-CoV-2 strains including the delta variant (B.1.617.2) (18). Our study thus provides indirect evidence about the clinico-demographic profile of Indian patients infected with the Omicron (B.1.1.529) variant, although formal variant testing was not done as a part of the study. On comparing mildly symptomatic and asymptomatic patients, a higher BMI (and a trend toward male predilection) was seen in symptomatic patients in our cohort, which is in line with the available literature (19). Counterintuitively, no difference in the presence of comorbidities, time since last vaccine dose, and past COVID infection was noted between symptomatic and asymptomatic patients.

The collected data show that multi-faceted issues of COVID-19 affected healthcare workers under home isolation can be managed remotely using telemedicine. The merits of the study are inclusion of microbiologically confirmed COVID-19 patients (rather than suspect patients), a focus on health care workers as the target population, and employment of a holistic remote monitoring model comprising of mandatory phone calls assisted by home oximetry, administration of a predesigned google form with relevant clinical details, and voluntary, opt-in software-based and/or telephonic consultation. Use of existing resources and staff (by virtue of a separate Department of Telemedicine) avoided issues pertaining to cost-effectiveness and sustainability of the model. The limitations of the study include a relatively small sample size from a single center; a lack of progression to moderate or severe disease was seen, which could be explained by the inherently mild nature of the Omicron (B.1.1.529) variant, small sample size, and inclusion of a fairly young study population with a relatively low prevalence of comorbidities. A formal assessment of the participants' psychological problems was not done. Analysis of factors associated with symptomatic COVID-19 included only limited variables. Inclusion of a control group could have facilitated better inferences regarding effectiveness of remote monitoring and the extent of improvement in patient outcomes. Finally, since the study population comprised of healthcare workers who are better informed about the disease, these findings cannot be directly extrapolated to the general population, although the principles of remote monitoring would still be valid.

## Conclusion

Integration and deployment of digital technology is important in the management of COVID-19. Diverse medical and psychosocial issues of COVID-19 patients under home isolation can be holistically managed remotely through telemedicine and digital technology. The clinico-demographic profile of healthcare workers infected with mild COVID-19 during the third wave [epidemiologically linked to Omicron (B.1.1.529) variant] has also been reported.

## Data Availability Statement

The original contributions presented in the study are included in the article/supplementary material, further inquiries can be directed to the corresponding author.

## Ethics Statement

The studies involving human participants were reviewed and approved by Institute Ethics Committee PGIMER Chandigarh INT/IEC/2022/SPL-281. The patients/participants provided their written informed consent to participate in this study.

## Author Contributions

SJ and AA planned the study and conducted the study. AB helped in gathering and entering the data. PL helped in providing the healthcare workers data who came COVID positive. AC and MaS helped in preparing final version of manuscript. MeS looked overall performance of the study and also helped in getting the ethical clearance of the study. All authors contributed to the article and approved the submitted version.

## Conflict of Interest

The authors declare that the research was conducted in the absence of any commercial or financial relationships that could be construed as a potential conflict of interest.

## Publisher's Note

All claims expressed in this article are solely those of the authors and do not necessarily represent those of their affiliated organizations, or those of the publisher, the editors and the reviewers. Any product that may be evaluated in this article, or claim that may be made by its manufacturer, is not guaranteed or endorsed by the publisher.
